# (*E*)-4-Meth­oxy-*N*′-(3,4,5-trimeth­oxy­benzyl­idene)benzohydrazide

**DOI:** 10.1107/S1600536812003534

**Published:** 2012-02-04

**Authors:** Hoong-Kun Fun, Premrudee Promdet, Jirapa Horkaew, Suchada Chantrapromma

**Affiliations:** a X-ray Crystallography Unit, School of Physics, Universiti Sains Malaysia, 11800 USM, Penang, Malaysia; b Crystal Materials Research Unit, Department of Chemistry, Faculty of Science, Prince of Songkla University, Hat-Yai, Songkhla 90112, Thailand

## Abstract

In the asymmetric unit of the title compound, C_18_H_20_N_2_O_5_, there are two crystallographic independent mol­ecules. Both mol­ecules are twisted; the dihedral angle between the two benzene rings is 7.2 (5)° in one mol­ecule, whereas it is 85.9 (4)° in the other. Of the three meth­oxy groups in the 3,4,5-trimeth­oxy­phenyl unit, two meth­oxy groups at *meta* positions are approximately coplanar with the benzene plane [C—O—C—C torsion angles of −2.3 (13)–4.8 (11)°], but the other meth­oxy, at the *para* position, is out of the plane [C—O—C—C of 72.8 (9)° in one mol­ecule and −77.5 (9)° in the other]. In the crystal, mol­ecules are linked by N—H⋯O hydrogen bonds and weak C—H⋯O inter­actions into tapes along the *b* axis. C—H⋯π inter­actions are also present.

## Related literature

For bond-length data, see: Allen *et al.* (1987 ▶). For related structures, see: Fun *et al.* (2011 ▶); Horkaew *et al.* (2011 ▶); Promdet *et al.* (2011 ▶). For background and applications of benzohydrazide derivatives, see: Angelusiu *et al.* (2010 ▶); Bedia *et al.* (2006 ▶); Loncle *et al.* (2004 ▶); Melnyk *et al.* (2006 ▶); Raj *et al.* (2007 ▶).
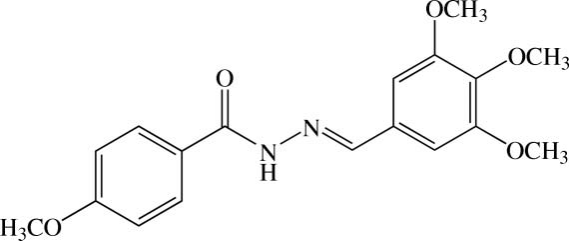


## Experimental

### 

#### Crystal data

C_18_H_20_N_2_O_5_
*M* *_r_* = 344.36
Monoclinic, 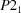

*a* = 13.3344 (17) Å
*b* = 5.0484 (6) Å
*c* = 25.767 (3) Å
β = 98.250 (2)°
*V* = 1716.6 (4) Å^3^
*Z* = 4
Mo *K*α radiation
μ = 0.10 mm^−1^
*T* = 297 K
0.26 × 0.25 × 0.11 mm

#### Data collection

Bruker SMART APEXII CCD area-detector diffractometer
Absorption correction: multi-scan (*SADABS*; Bruker, 2005 ▶) *T*_min_ = 0.975, *T*_max_ = 0.990
12488 measured reflections
3361 independent reflections
2671 reflections with *I* > 2σ(*I*)
*R*_int_ = 0.039

#### Refinement

*R*[*F* ^2^ > 2σ(*F* ^2^)] = 0.090
*wR*(*F* ^2^) = 0.275
*S* = 1.05
3361 reflections
453 parameters
1 restraint
H-atom parameters constrained
Δρ_max_ = 0.75 e Å^−3^
Δρ_min_ = −0.47 e Å^−3^

### 

Data collection: *APEX2* (Bruker, 2005 ▶); cell refinement: *SAINT* (Bruker, 2005 ▶); data reduction: *SAINT*; program(s) used to solve structure: *SHELXTL* (Sheldrick, 2008 ▶); program(s) used to refine structure: *SHELXTL*; molecular graphics: *SHELXTL*; software used to prepare material for publication: *SHELXTL* and *PLATON* (Spek, 2009 ▶).

## Supplementary Material

Crystal structure: contains datablock(s) global, I. DOI: 10.1107/S1600536812003534/is5059sup1.cif

Structure factors: contains datablock(s) I. DOI: 10.1107/S1600536812003534/is5059Isup2.hkl

Supplementary material file. DOI: 10.1107/S1600536812003534/is5059Isup3.cml

Additional supplementary materials:  crystallographic information; 3D view; checkCIF report

## Figures and Tables

**Table 1:** Hydrogen-bond geometry (Å, °) *Cg*1 and *Cg*2 are the centroids of the C9*A*–C14*A* and C9*B*–C14*B* rings, respectively.

*D*—H⋯*A*	*D*—H	H⋯*A*	*D*⋯*A*	*D*—H⋯*A*
N1*A*—H1*NA*⋯O1*A*^i^	0.86	2.11	2.944 (13)	164
N1*B*—H1*NB*⋯O1*B*^i^	0.85	2.21	2.939 (9)	144
C8*B*—H8*B*⋯O1*B*^i^	0.93	2.52	3.287 (11)	140
C15*B*—H15*D*⋯O2*A*^ii^	0.96	2.60	3.498 (14)	156
C17*B*—H17*D*⋯O4*B*^iii^	0.96	2.60	3.420 (10)	144
C16*A*—H16*B*⋯*Cg*1^iv^	0.96	2.66	3.429 (10)	138
C16*B*—H16*F*⋯*Cg*2^iv^	0.96	2.77	3.697 (10)	162
C18*A*—H18*B*⋯*Cg*1^i^	0.96	2.85	3.739 (10)	155
C18*B*—H18*F*⋯*Cg*2^i^	0.96	2.74	3.583 (10)	146

Symmetry codes: (i) 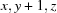; (ii) 
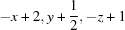
; (iii) 
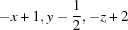
; (iv) 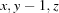.

**Fig. 1.**
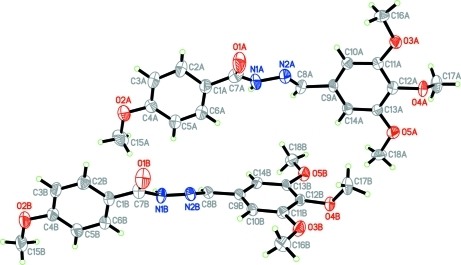
The molecular structure of the title compound, showing 40% probability displacement ellipsoids and the atom-numbering scheme.

**Fig. 2.**
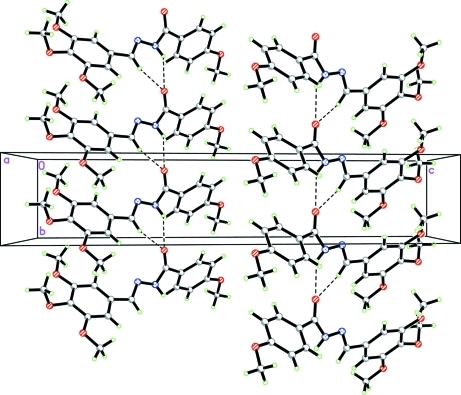
The crystal packing of the title compound viewed along the a axis, showing tapes running along the b axis. Hydrogen bonds were drawn as dashed lines.

## supplementary crystallographic information

### Comment

Benzohydrazide derivatives obtained from the reaction of aldehyde with hydrazine have been demonstrated to possess excellent biological properties such as antibacterial (Angelusiu *et al.*, 2010) antifungal (Loncle *et al.*, 2004), antimalarial (Melnyk *et al.*, 2006) and antiproliferative (Raj *et al.*, 2007) activities. The title benzohydrazide (I) is the condensation product of 4-methoxybenzohydrazide and 3,4,5-trimethoxybenzaldehyde which was synthesized in order to study and compare its biological properties with other related compounds (Fun *et al.*, 2011; Horkaew *et al.*, 2011; Promdet *et al.*, 2011). (I) was screened for antioxidant activities and found to be inactive.

The title compound (Fig. 1) crystallized out with two crystallographically independent molecules *A* and *B* per asymmetric unit with the two molecules having slight differences in bond angles. The molecule exists in a *trans*-configuration with respect to the C8═N2 bond [1.243 (12) Å in molecule *A* and 1.290 (11) Å in molecule *B*] and the torsion angle N1–N2–C8–C9 = -174.4 (7)° for molecule *A* [-172.2 (6)° for molecule *B*]. Molecule *A* is less twisted than molecule *B* which is indicated by the dihedral angle between the two benzene rings being 7.2 (5)° in molecule *A* whereas it is 85.9 (4)° in molecule *B*. The middle bridge fragment (O1/C7/N1/N2/C8) of molecule *A* is also less twisted than that of molecule *B* with the dihedral angle between the mean planes of O1/C7/N1 and N1/N2/C8 being 8.3 (15)° in molecule *A* whereas it is 26.0 (11)° in molecule *B*. The methoxy group of 4-methoxyphenyl (at atom C4) is co-planar with its bound benzene ring [torsion angle C15–O2–C4–C5 = 1.16 (18)° and the *r.m.s* 0.0222 (9) Å in molecule *A* [-4.3 (15)° and *r.m.s* 0.0164 (9) Å in molecule *B*] for the eight non-H atoms. The three methoxy substituents of 3,4,5-trimethoxyphenyl unit have two different orientations in which the two methoxy groups at two *meta*-positions or at atoms C11 and C13 are co-planar with torsion angles C16–O3–C11–C10 = 4.8 (11)° and C18–O5–C13–C12 = 178.5 (7)°, whereas the third one at the *para*-position or at atom C12 is tilted out of plane with the torsion angle C17–O4–C12–C11 = 72.8 (9)° [the corresponding values are -2.3, 179.2 (8) and -77.5 (9)°, respectively, in molecule *B*]. Bond distances are of normal values (Allen *et al.*, 1987) and are comparable with the related structures (Fun *et al.*, 2011; Horkaew *et al.*, 2011; Promdet *et al.*, 2011). In the crystal packing (Fig. 2), the molecules are linked by N—H···O hydrogen bonds and weak C—H···O interactions (Table 1) into tapes along the *b* axis. C—H···π weak interactions were presented (Table 1).

### Experimental

The title compound (I) was prepared by dissolving 4-methoxybenzohydrazide (2 mmol, 0.30 g) in ethanol (10 ml). The solution of 3,4,5-trimethoxybenzaldehyde (2 mmol, 0.40 g) in ethanol (10 ml) was then added slowly to the reaction. The mixture was refluxed for around 6 hr. The solution was then cooled to room temperature and evaporated by reduced pressure. Colorless block-shaped single crystals of the title compound suitable for *X*-ray structure determination were recrystallized from methanol by slow evaporation of the solvent at room temperature after several days (m.p. 464-465 K).

### Refinement

All H atoms were positioned geometrically and allowed to ride on their parent atoms, with d(N—H) = 0.85–0.86 Å, and d(C—H) = 0.93 Å for aromatic and CH and 0.96 Å for CH_3_ atoms. The *U*_iso_(H) values were constrained to be 1.5*U*_eq_ of the carrier atom for methyl H atoms and 1.2*U*_eq_ for the remaining H atoms. A rotating group model was used for the methyl groups. A total of 2541 Friedel pairs were merged before final refinement as there is no large anomalous dispersion for the determination of the absolute configuration.

### Crystal data

**Table:**

C_18_H_20_N_2_O_5_	*F*(000) = 728
*M**_r_* = 344.36	*D*_x_ = 1.332 Mg m^−^^3^
Monoclinic, *P*2_1_	Melting point = 464–465 K
Hall symbol: P 2yb	Mo *K*α radiation, λ = 0.71073 Å
*a* = 13.3344 (17) Å	Cell parameters from 3361 reflections
*b* = 5.0484 (6) Å	θ = 1.6–25.0°
*c* = 25.767 (3) Å	µ = 0.10 mm^−^^1^
β = 98.250 (2)°	*T* = 297 K
*V* = 1716.6 (4) Å^3^	Block, colorless
*Z* = 4	0.26 × 0.25 × 0.11 mm

### Data collection

**Table:**

Bruker SMART APEXII CCD area-detector diffractometer	3361 independent reflections
Radiation source: fine-focus sealed tube	2671 reflections with *I* > 2σ(*I*)
Graphite monochromator	*R*_int_ = 0.039
Detector resolution: 8.33 pixels mm^-1^	θ_max_ = 25.0°, θ_min_ = 1.6°
ω scans	*h* = −15→15
Absorption correction: multi-scan (*SADABS*; Bruker, 2005)	*k* = −6→6
*T*_min_ = 0.975, *T*_max_ = 0.990	*l* = −30→30
12488 measured reflections	

### Refinement

**Table:**

Refinement on *F*^2^	Primary atom site location: structure-invariant direct methods
Least-squares matrix: full	Secondary atom site location: difference Fourier map
*R*[*F*^2^ > 2σ(*F*^2^)] = 0.090	Hydrogen site location: inferred from neighbouring sites
*wR*(*F*^2^) = 0.275	H-atom parameters constrained
*S* = 1.05	*w* = 1/[σ^2^(*F*_o_^2^) + (0.1357*P*)^2^ + 4.5571*P*] where *P* = (*F*_o_^2^ + 2*F*_c_^2^)/3
3361 reflections	(Δ/σ)_max_ = 0.001
453 parameters	Δρ_max_ = 0.75 e Å^−^^3^
1 restraint	Δρ_min_ = −0.47 e Å^−^^3^

### Special details

**Table:**

Geometry. All esds (except the esd in the dihedral angle between two l.s. planes) are estimated using the full covariance matrix. The cell esds are taken into account individually in the estimation of esds in distances, angles and torsion angles; correlations between esds in cell parameters are only used when they are defined by crystal symmetry. An approximate (isotropic) treatment of cell esds is used for estimating esds involving l.s. planes.
Refinement. Refinement of F^2^ against ALL reflections. The weighted R-factor wR and goodness of fit S are based on F^2^, conventional R-factors R are based on F, with F set to zero for negative F^2^. The threshold expression of F^2^ > 2sigma(F^2^) is used only for calculating R-factors(gt) etc. and is not relevant to the choice of reflections for refinement. R-factors based on F^2^ are statistically about twice as large as those based on F, and R- factors based on ALL data will be even larger.

### Fractional atomic coordinates and isotropic or equivalent isotropic displacement parameters (Å^2^)

**Table:**

	*x*	*y*	*z*	*U*_iso_*/*U*_eq_	
O1A	0.3343 (7)	−0.3991 (18)	0.6742 (3)	0.078 (2)	
O2A	0.6531 (5)	0.112 (2)	0.5437 (3)	0.080 (3)	
O3A	−0.0197 (4)	−0.2248 (13)	0.8547 (2)	0.0443 (14)	
O4A	0.0405 (4)	0.0695 (13)	0.93724 (19)	0.0450 (14)	
O5A	0.1905 (4)	0.4311 (15)	0.9377 (2)	0.0543 (17)	
N1A	0.3208 (5)	0.0316 (19)	0.6969 (3)	0.055 (2)	
H1NA	0.3373	0.1922	0.6909	0.066*	
N2A	0.2568 (5)	−0.0252 (18)	0.7341 (3)	0.053 (2)	
C1A	0.4361 (7)	−0.083 (2)	0.6382 (3)	0.053 (2)	
C2A	0.4404 (8)	−0.243 (4)	0.5919 (4)	0.101 (4)	
H2A	0.4008	−0.3925	0.5830	0.121*	
C3A	0.5149 (8)	−0.139 (4)	0.5609 (4)	0.101 (4)	
H3A	0.5156	−0.2111	0.5278	0.121*	
C4A	0.5852 (6)	0.060 (3)	0.5768 (3)	0.063 (3)	
C5A	0.5800 (7)	0.192 (3)	0.6231 (3)	0.065 (3)	
H5A	0.6257	0.3269	0.6340	0.078*	
C6A	0.5070 (7)	0.123 (3)	0.6530 (4)	0.077 (4)	
H6A	0.5037	0.2148	0.6841	0.093*	
C7A	0.3549 (8)	−0.1670 (18)	0.6713 (4)	0.054 (2)	
C8A	0.2406 (7)	0.1708 (19)	0.7608 (3)	0.045 (2)	
H8AA	0.2653	0.3358	0.7528	0.054*	
C9A	0.1820 (6)	0.1442 (17)	0.8058 (3)	0.0377 (19)	
C10A	0.1069 (5)	−0.0388 (17)	0.8060 (3)	0.0376 (18)	
H10A	0.0892	−0.1480	0.7771	0.045*	
C11A	0.0568 (5)	−0.0620 (15)	0.8494 (3)	0.0321 (16)	
C12A	0.0869 (6)	0.1012 (18)	0.8933 (3)	0.0373 (18)	
C13A	0.1640 (5)	0.2884 (17)	0.8925 (3)	0.0337 (17)	
C14A	0.2105 (5)	0.3152 (17)	0.8482 (3)	0.0364 (18)	
H14A	0.2597	0.4442	0.8466	0.044*	
C15A	0.7253 (8)	0.309 (3)	0.5574 (4)	0.086 (4)	
H15C	0.7754	0.3023	0.5342	0.129*	
H15B	0.7573	0.2815	0.5928	0.129*	
H15A	0.6929	0.4796	0.5547	0.129*	
C16A	−0.0507 (6)	−0.413 (2)	0.8128 (3)	0.048 (2)	
H16C	−0.0986	−0.5353	0.8237	0.072*	
H16B	0.0075	−0.5075	0.8048	0.072*	
H16A	−0.0817	−0.3192	0.7822	0.072*	
C17A	−0.0268 (7)	0.284 (2)	0.9456 (4)	0.058 (3)	
H17C	−0.0591	0.2464	0.9758	0.088*	
H17B	−0.0775	0.3028	0.9153	0.088*	
H17A	0.0112	0.4454	0.9512	0.088*	
C18A	0.2695 (6)	0.619 (2)	0.9396 (4)	0.056 (3)	
H18C	0.2762	0.7116	0.9724	0.085*	
H18B	0.2542	0.7425	0.9113	0.085*	
H18A	0.3319	0.5292	0.9364	0.085*	
O1B	0.8368 (6)	−0.3520 (12)	0.6791 (3)	0.064 (2)	
O2B	1.1551 (5)	0.082 (2)	0.5408 (2)	0.071 (2)	
O3B	0.6942 (4)	−0.0861 (15)	0.9327 (2)	0.0526 (16)	
O4B	0.5447 (4)	0.2633 (13)	0.93709 (19)	0.0432 (14)	
O5B	0.4755 (4)	0.5755 (13)	0.8561 (2)	0.0453 (14)	
N1B	0.8346 (5)	0.0774 (14)	0.7016 (2)	0.0397 (15)	
H1NB	0.8270	0.2117	0.6812	0.048*	
N2B	0.7795 (5)	0.0341 (15)	0.7419 (2)	0.0402 (16)	
C1B	0.9427 (6)	−0.0541 (16)	0.6400 (3)	0.0367 (17)	
C2B	0.9451 (7)	−0.1879 (19)	0.5939 (3)	0.051 (2)	
H2B	0.8949	−0.3130	0.5839	0.061*	
C3B	1.0160 (7)	−0.149 (2)	0.5623 (3)	0.053 (2)	
H3B	1.0173	−0.2531	0.5326	0.064*	
C4B	1.0878 (6)	0.052 (2)	0.5750 (3)	0.052 (2)	
C5B	1.0874 (6)	0.195 (2)	0.6201 (3)	0.048 (2)	
H5B	1.1358	0.3256	0.6293	0.058*	
C6B	1.0139 (6)	0.143 (2)	0.6523 (3)	0.047 (2)	
H6B	1.0129	0.2431	0.6825	0.056*	
C7B	0.8691 (7)	−0.1257 (18)	0.6757 (3)	0.045 (2)	
C8B	0.7321 (5)	0.241 (2)	0.7552 (3)	0.0399 (19)	
H8B	0.7299	0.3951	0.7354	0.048*	
C9B	0.6815 (5)	0.2261 (18)	0.8023 (3)	0.0395 (19)	
C10B	0.7130 (5)	0.061 (2)	0.8438 (3)	0.0406 (19)	
H10B	0.7669	−0.0535	0.8417	0.049*	
C11B	0.6668 (5)	0.0605 (19)	0.8886 (3)	0.0392 (18)	
C12B	0.5874 (5)	0.2440 (18)	0.8921 (3)	0.0340 (17)	
C13B	0.5554 (5)	0.4060 (18)	0.8500 (3)	0.0348 (17)	
C14B	0.6028 (5)	0.4086 (19)	0.8057 (3)	0.0399 (19)	
H14B	0.5832	0.5282	0.7787	0.048*	
C15B	1.2275 (8)	0.293 (3)	0.5513 (4)	0.086 (4)	
H15D	1.2581	0.3281	0.5204	0.129*	
H15E	1.2790	0.2425	0.5795	0.129*	
H15F	1.1939	0.4499	0.5609	0.129*	
C16B	0.7757 (6)	−0.263 (2)	0.9328 (4)	0.052 (2)	
H16D	0.7973	−0.3246	0.9679	0.078*	
H16E	0.8309	−0.1749	0.9199	0.078*	
H16F	0.7544	−0.4118	0.9106	0.078*	
C17B	0.4744 (7)	0.062 (2)	0.9456 (3)	0.059 (3)	
H17D	0.4641	0.0629	0.9817	0.088*	
H17E	0.5006	−0.1078	0.9372	0.088*	
H17F	0.4111	0.0932	0.9237	0.088*	
C18B	0.4404 (6)	0.746 (2)	0.8154 (3)	0.050 (2)	
H18D	0.3865	0.8527	0.8252	0.075*	
H18E	0.4158	0.6453	0.7846	0.075*	
H18F	0.4947	0.8590	0.8082	0.075*	

### Atomic displacement parameters (Å^2^)

**Table:**

	*U*^11^	*U*^22^	*U*^33^	*U*^12^	*U*^13^	*U*^23^
O1A	0.111 (6)	0.058 (5)	0.076 (5)	−0.016 (5)	0.054 (4)	−0.001 (4)
O2A	0.064 (4)	0.122 (8)	0.058 (4)	−0.021 (5)	0.028 (3)	−0.003 (5)
O3A	0.047 (3)	0.042 (3)	0.046 (3)	−0.009 (3)	0.014 (3)	−0.004 (3)
O4A	0.060 (3)	0.045 (3)	0.036 (3)	0.007 (3)	0.023 (2)	0.011 (3)
O5A	0.058 (3)	0.061 (4)	0.045 (3)	−0.017 (4)	0.010 (3)	−0.010 (3)
N1A	0.055 (4)	0.064 (5)	0.053 (4)	−0.006 (4)	0.028 (3)	0.007 (4)
N2A	0.052 (4)	0.061 (6)	0.048 (4)	−0.005 (4)	0.017 (3)	−0.001 (4)
C1A	0.056 (5)	0.058 (6)	0.047 (5)	−0.007 (5)	0.017 (4)	−0.003 (5)
C2A	0.076 (5)	0.179 (12)	0.052 (4)	−0.006 (7)	0.025 (4)	−0.037 (7)
C3A	0.076 (5)	0.179 (12)	0.052 (4)	−0.006 (7)	0.025 (4)	−0.037 (7)
C4A	0.043 (4)	0.101 (9)	0.047 (5)	−0.002 (6)	0.009 (4)	−0.002 (6)
C5A	0.055 (5)	0.095 (9)	0.049 (5)	−0.002 (6)	0.015 (4)	−0.013 (6)
C6A	0.059 (6)	0.128 (12)	0.047 (5)	−0.001 (7)	0.014 (5)	−0.014 (7)
C7A	0.070 (6)	0.029 (5)	0.061 (5)	0.017 (5)	0.006 (5)	−0.016 (4)
C8A	0.057 (5)	0.047 (5)	0.034 (4)	0.007 (4)	0.014 (4)	0.000 (4)
C9A	0.038 (4)	0.042 (5)	0.036 (4)	0.007 (4)	0.014 (3)	−0.002 (4)
C10A	0.036 (4)	0.038 (5)	0.039 (4)	0.007 (4)	0.007 (3)	−0.002 (4)
C11A	0.035 (4)	0.022 (4)	0.039 (4)	0.005 (3)	0.008 (3)	0.006 (3)
C12A	0.043 (4)	0.034 (4)	0.036 (4)	0.010 (4)	0.009 (3)	0.005 (4)
C13A	0.030 (3)	0.034 (4)	0.037 (4)	0.009 (3)	0.005 (3)	−0.005 (4)
C14A	0.033 (4)	0.036 (5)	0.042 (4)	0.007 (4)	0.010 (3)	0.001 (4)
C15A	0.066 (6)	0.115 (12)	0.081 (7)	−0.029 (8)	0.023 (6)	0.027 (8)
C16A	0.048 (4)	0.044 (5)	0.050 (5)	−0.002 (5)	−0.002 (4)	−0.010 (5)
C17A	0.076 (6)	0.045 (6)	0.063 (5)	0.009 (5)	0.038 (5)	0.001 (5)
C18A	0.050 (5)	0.061 (6)	0.054 (5)	−0.009 (5)	−0.006 (4)	−0.015 (5)
O1B	0.100 (6)	0.023 (3)	0.076 (5)	−0.001 (3)	0.039 (4)	0.005 (3)
O2B	0.063 (4)	0.104 (6)	0.052 (3)	0.008 (5)	0.028 (3)	0.001 (5)
O3B	0.060 (3)	0.064 (4)	0.035 (3)	0.018 (4)	0.010 (3)	0.010 (3)
O4B	0.050 (3)	0.046 (3)	0.037 (3)	−0.001 (3)	0.018 (2)	0.000 (3)
O5B	0.049 (3)	0.044 (3)	0.045 (3)	0.021 (3)	0.013 (2)	0.004 (3)
N1B	0.056 (4)	0.030 (4)	0.037 (3)	0.010 (3)	0.020 (3)	0.001 (3)
N2B	0.042 (3)	0.040 (4)	0.041 (3)	−0.004 (3)	0.015 (3)	0.007 (3)
C1B	0.044 (4)	0.025 (4)	0.044 (4)	0.013 (3)	0.015 (3)	0.006 (3)
C2B	0.060 (5)	0.043 (5)	0.051 (5)	−0.008 (5)	0.012 (4)	−0.010 (4)
C3B	0.059 (5)	0.057 (6)	0.046 (5)	−0.001 (5)	0.021 (4)	−0.015 (4)
C4B	0.047 (4)	0.072 (7)	0.038 (4)	0.007 (5)	0.008 (3)	0.000 (5)
C5B	0.047 (5)	0.051 (6)	0.049 (5)	−0.002 (4)	0.014 (4)	0.002 (4)
C6B	0.047 (5)	0.051 (6)	0.043 (4)	0.003 (4)	0.013 (4)	−0.006 (4)
C7B	0.057 (5)	0.030 (5)	0.051 (5)	0.008 (4)	0.016 (4)	0.007 (4)
C8B	0.035 (4)	0.046 (5)	0.040 (4)	−0.003 (4)	0.011 (3)	−0.001 (4)
C9B	0.034 (4)	0.044 (5)	0.041 (4)	−0.005 (4)	0.008 (3)	−0.009 (4)
C10B	0.034 (4)	0.047 (5)	0.042 (4)	0.009 (4)	0.011 (3)	−0.009 (4)
C11B	0.039 (4)	0.038 (4)	0.040 (4)	0.006 (4)	0.004 (3)	−0.001 (4)
C12B	0.034 (4)	0.038 (4)	0.032 (4)	0.006 (4)	0.010 (3)	−0.002 (4)
C13B	0.030 (3)	0.040 (4)	0.037 (4)	0.001 (4)	0.013 (3)	−0.010 (4)
C14B	0.040 (4)	0.048 (5)	0.032 (4)	0.002 (4)	0.007 (3)	−0.003 (4)
C15B	0.061 (6)	0.124 (12)	0.081 (7)	−0.032 (8)	0.040 (6)	−0.035 (9)
C16B	0.052 (5)	0.038 (5)	0.063 (5)	0.012 (4)	0.004 (4)	0.010 (5)
C17B	0.072 (6)	0.060 (6)	0.053 (5)	−0.002 (6)	0.033 (5)	0.008 (6)
C18B	0.047 (5)	0.051 (5)	0.051 (5)	0.015 (5)	0.004 (4)	−0.009 (5)

### Geometric parameters (Å, º)

**Table:**

O1A—C7A	1.208 (13)	O1B—C7B	1.229 (11)
O2A—C4A	1.355 (10)	O2B—C4B	1.354 (9)
O2A—C15A	1.394 (15)	O2B—C15B	1.438 (15)
O3A—C11A	1.333 (9)	O3B—C11B	1.361 (10)
O3A—C16A	1.452 (10)	O3B—C16B	1.408 (10)
O4A—C12A	1.376 (8)	O4B—C12B	1.366 (8)
O4A—C17A	1.442 (11)	O4B—C17B	1.422 (11)
O5A—C13A	1.371 (9)	O5B—C18B	1.386 (11)
O5A—C18A	1.412 (11)	O5B—C13B	1.394 (9)
N1A—C7A	1.315 (12)	N1B—C7B	1.340 (11)
N1A—N2A	1.401 (9)	N1B—N2B	1.374 (8)
N1A—H1NA	0.8600	N1B—H1NB	0.8545
N2A—C8A	1.243 (12)	N2B—C8B	1.290 (11)
C1A—C6A	1.422 (17)	C1B—C2B	1.371 (11)
C1A—C2A	1.449 (16)	C1B—C6B	1.380 (12)
C1A—C7A	1.532 (13)	C1B—C7B	1.483 (11)
C2A—C3A	1.459 (18)	C2B—C3B	1.349 (11)
C2A—H2A	0.9300	C2B—H2B	0.9300
C3A—C4A	1.39 (2)	C3B—C4B	1.402 (14)
C3A—H3A	0.9300	C3B—H3B	0.9300
C4A—C5A	1.377 (14)	C4B—C5B	1.367 (12)
C5A—C6A	1.370 (13)	C5B—C6B	1.395 (11)
C5A—H5A	0.9300	C5B—H5B	0.9300
C6A—H6A	0.9300	C6B—H6B	0.9300
C8A—C9A	1.493 (10)	C8B—C9B	1.474 (10)
C8A—H8AA	0.9300	C8B—H8B	0.9300
C9A—C10A	1.363 (11)	C9B—C10B	1.371 (12)
C9A—C14A	1.403 (11)	C9B—C14B	1.408 (11)
C10A—C11A	1.388 (10)	C10B—C11B	1.385 (10)
C10A—H10A	0.9300	C10B—H10B	0.9300
C11A—C12A	1.409 (11)	C11B—C12B	1.420 (11)
C12A—C13A	1.398 (11)	C12B—C13B	1.376 (11)
C13A—C14A	1.381 (10)	C13B—C14B	1.382 (9)
C14A—H14A	0.9300	C14B—H14B	0.9300
C15A—H15C	0.9600	C15B—H15D	0.9600
C15A—H15B	0.9600	C15B—H15E	0.9600
C15A—H15A	0.9600	C15B—H15F	0.9600
C16A—H16C	0.9600	C16B—H16D	0.9600
C16A—H16B	0.9600	C16B—H16E	0.9600
C16A—H16A	0.9600	C16B—H16F	0.9600
C17A—H17C	0.9600	C17B—H17D	0.9600
C17A—H17B	0.9600	C17B—H17E	0.9600
C17A—H17A	0.9600	C17B—H17F	0.9600
C18A—H18C	0.9600	C18B—H18D	0.9600
C18A—H18B	0.9600	C18B—H18E	0.9600
C18A—H18A	0.9600	C18B—H18F	0.9600
			
C4A—O2A—C15A	118.7 (9)	C4B—O2B—C15B	116.7 (8)
C11A—O3A—C16A	117.9 (6)	C11B—O3B—C16B	117.5 (6)
C12A—O4A—C17A	113.5 (6)	C12B—O4B—C17B	116.3 (7)
C13A—O5A—C18A	118.9 (6)	C18B—O5B—C13B	118.5 (6)
C7A—N1A—N2A	118.3 (9)	C7B—N1B—N2B	120.9 (7)
C7A—N1A—H1NA	120.8	C7B—N1B—H1NB	108.7
N2A—N1A—H1NA	120.8	N2B—N1B—H1NB	124.3
C8A—N2A—N1A	112.9 (8)	C8B—N2B—N1B	114.0 (7)
C6A—C1A—C2A	121.8 (9)	C2B—C1B—C6B	116.9 (7)
C6A—C1A—C7A	123.3 (8)	C2B—C1B—C7B	121.2 (8)
C2A—C1A—C7A	114.9 (10)	C6B—C1B—C7B	121.9 (8)
C1A—C2A—C3A	111.1 (15)	C3B—C2B—C1B	123.9 (9)
C1A—C2A—H2A	124.5	C3B—C2B—H2B	118.0
C3A—C2A—H2A	124.5	C1B—C2B—H2B	118.0
C4A—C3A—C2A	125.6 (11)	C2B—C3B—C4B	118.6 (8)
C4A—C3A—H3A	117.2	C2B—C3B—H3B	120.7
C2A—C3A—H3A	117.2	C4B—C3B—H3B	120.7
O2A—C4A—C5A	124.9 (11)	O2B—C4B—C5B	125.4 (10)
O2A—C4A—C3A	116.0 (9)	O2B—C4B—C3B	114.9 (8)
C5A—C4A—C3A	119.1 (9)	C5B—C4B—C3B	119.6 (8)
C6A—C5A—C4A	119.6 (11)	C4B—C5B—C6B	119.7 (9)
C6A—C5A—H5A	120.2	C4B—C5B—H5B	120.2
C4A—C5A—H5A	120.2	C6B—C5B—H5B	120.2
C5A—C6A—C1A	122.2 (10)	C1B—C6B—C5B	121.2 (8)
C5A—C6A—H6A	118.9	C1B—C6B—H6B	119.4
C1A—C6A—H6A	118.9	C5B—C6B—H6B	119.4
O1A—C7A—N1A	127.7 (10)	O1B—C7B—N1B	121.8 (8)
O1A—C7A—C1A	119.1 (9)	O1B—C7B—C1B	122.8 (8)
N1A—C7A—C1A	113.0 (9)	N1B—C7B—C1B	115.3 (8)
N2A—C8A—C9A	120.8 (8)	N2B—C8B—C9B	118.5 (8)
N2A—C8A—H8AA	119.6	N2B—C8B—H8B	120.7
C9A—C8A—H8AA	119.6	C9B—C8B—H8B	120.7
C10A—C9A—C14A	122.1 (7)	C10B—C9B—C14B	119.8 (7)
C10A—C9A—C8A	122.1 (7)	C10B—C9B—C8B	123.1 (7)
C14A—C9A—C8A	115.9 (7)	C14B—C9B—C8B	116.8 (8)
C9A—C10A—C11A	120.0 (7)	C9B—C10B—C11B	121.8 (7)
C9A—C10A—H10A	120.0	C9B—C10B—H10B	119.1
C11A—C10A—H10A	120.0	C11B—C10B—H10B	119.1
O3A—C11A—C10A	126.8 (7)	O3B—C11B—C10B	126.8 (7)
O3A—C11A—C12A	114.4 (6)	O3B—C11B—C12B	114.5 (6)
C10A—C11A—C12A	118.8 (7)	C10B—C11B—C12B	118.4 (7)
O4A—C12A—C13A	120.6 (7)	O4B—C12B—C13B	120.5 (7)
O4A—C12A—C11A	118.8 (7)	O4B—C12B—C11B	120.4 (7)
C13A—C12A—C11A	120.6 (7)	C13B—C12B—C11B	119.2 (6)
O5A—C13A—C14A	123.9 (7)	C12B—C13B—C14B	122.0 (7)
O5A—C13A—C12A	116.4 (6)	C12B—C13B—O5B	115.6 (6)
C14A—C13A—C12A	119.7 (7)	C14B—C13B—O5B	122.4 (7)
C13A—C14A—C9A	118.7 (7)	C13B—C14B—C9B	118.5 (8)
C13A—C14A—H14A	120.6	C13B—C14B—H14B	120.7
C9A—C14A—H14A	120.6	C9B—C14B—H14B	120.7
O2A—C15A—H15C	109.5	O2B—C15B—H15D	109.5
O2A—C15A—H15B	109.5	O2B—C15B—H15E	109.5
H15C—C15A—H15B	109.5	H15D—C15B—H15E	109.5
O2A—C15A—H15A	109.5	O2B—C15B—H15F	109.5
H15C—C15A—H15A	109.5	H15D—C15B—H15F	109.5
H15B—C15A—H15A	109.5	H15E—C15B—H15F	109.5
O3A—C16A—H16C	109.5	O3B—C16B—H16D	109.5
O3A—C16A—H16B	109.5	O3B—C16B—H16E	109.5
H16C—C16A—H16B	109.5	H16D—C16B—H16E	109.5
O3A—C16A—H16A	109.5	O3B—C16B—H16F	109.5
H16C—C16A—H16A	109.5	H16D—C16B—H16F	109.5
H16B—C16A—H16A	109.5	H16E—C16B—H16F	109.5
O4A—C17A—H17C	109.5	O4B—C17B—H17D	109.5
O4A—C17A—H17B	109.5	O4B—C17B—H17E	109.5
H17C—C17A—H17B	109.5	H17D—C17B—H17E	109.5
O4A—C17A—H17A	109.5	O4B—C17B—H17F	109.5
H17C—C17A—H17A	109.5	H17D—C17B—H17F	109.5
H17B—C17A—H17A	109.5	H17E—C17B—H17F	109.5
O5A—C18A—H18C	109.5	O5B—C18B—H18D	109.5
O5A—C18A—H18B	109.5	O5B—C18B—H18E	109.5
H18C—C18A—H18B	109.5	H18D—C18B—H18E	109.5
O5A—C18A—H18A	109.5	O5B—C18B—H18F	109.5
H18C—C18A—H18A	109.5	H18D—C18B—H18F	109.5
H18B—C18A—H18A	109.5	H18E—C18B—H18F	109.5
			
C7A—N1A—N2A—C8A	170.6 (9)	C7B—N1B—N2B—C8B	−165.1 (8)
C6A—C1A—C2A—C3A	−7.8 (19)	C6B—C1B—C2B—C3B	4.3 (14)
C7A—C1A—C2A—C3A	176.2 (11)	C7B—C1B—C2B—C3B	−173.6 (9)
C1A—C2A—C3A—C4A	10 (2)	C1B—C2B—C3B—C4B	−4.5 (15)
C15A—O2A—C4A—C5A	1.6 (18)	C15B—O2B—C4B—C5B	−4.3 (15)
C15A—O2A—C4A—C3A	−180.0 (12)	C15B—O2B—C4B—C3B	177.6 (9)
C2A—C3A—C4A—O2A	174.8 (15)	C2B—C3B—C4B—O2B	−178.8 (9)
C2A—C3A—C4A—C5A	−7 (2)	C2B—C3B—C4B—C5B	3.0 (14)
O2A—C4A—C5A—C6A	179.2 (12)	O2B—C4B—C5B—C6B	−179.6 (9)
C3A—C4A—C5A—C6A	0.9 (18)	C3B—C4B—C5B—C6B	−1.6 (14)
C4A—C5A—C6A—C1A	0.6 (18)	C2B—C1B—C6B—C5B	−2.6 (12)
C2A—C1A—C6A—C5A	3.4 (19)	C7B—C1B—C6B—C5B	175.2 (8)
C7A—C1A—C6A—C5A	179.0 (11)	C4B—C5B—C6B—C1B	1.4 (14)
N2A—N1A—C7A—O1A	3.5 (16)	N2B—N1B—C7B—O1B	15.2 (14)
N2A—N1A—C7A—C1A	−171.2 (7)	N2B—N1B—C7B—C1B	−169.2 (6)
C6A—C1A—C7A—O1A	−142.0 (12)	C2B—C1B—C7B—O1B	29.8 (14)
C2A—C1A—C7A—O1A	33.9 (15)	C6B—C1B—C7B—O1B	−148.0 (10)
C6A—C1A—C7A—N1A	33.2 (14)	C2B—C1B—C7B—N1B	−145.7 (9)
C2A—C1A—C7A—N1A	−150.8 (11)	C6B—C1B—C7B—N1B	36.5 (11)
N1A—N2A—C8A—C9A	−174.4 (7)	N1B—N2B—C8B—C9B	−172.2 (6)
N2A—C8A—C9A—C10A	−31.3 (13)	N2B—C8B—C9B—C10B	27.5 (12)
N2A—C8A—C9A—C14A	147.3 (9)	N2B—C8B—C9B—C14B	−158.0 (7)
C14A—C9A—C10A—C11A	−0.7 (12)	C14B—C9B—C10B—C11B	2.6 (13)
C8A—C9A—C10A—C11A	177.8 (7)	C8B—C9B—C10B—C11B	177.0 (8)
C16A—O3A—C11A—C10A	4.8 (11)	C16B—O3B—C11B—C10B	−2.3 (13)
C16A—O3A—C11A—C12A	−175.8 (7)	C16B—O3B—C11B—C12B	−177.2 (8)
C9A—C10A—C11A—O3A	177.8 (7)	C9B—C10B—C11B—O3B	−177.2 (8)
C9A—C10A—C11A—C12A	−1.7 (11)	C9B—C10B—C11B—C12B	−2.5 (13)
C17A—O4A—C12A—C13A	72.8 (9)	C17B—O4B—C12B—C13B	103.9 (10)
C17A—O4A—C12A—C11A	−108.5 (9)	C17B—O4B—C12B—C11B	−77.5 (9)
O3A—C11A—C12A—O4A	3.5 (10)	O3B—C11B—C12B—O4B	0.1 (12)
C10A—C11A—C12A—O4A	−177.0 (7)	C10B—C11B—C12B—O4B	−175.3 (8)
O3A—C11A—C12A—C13A	−177.7 (7)	O3B—C11B—C12B—C13B	178.6 (8)
C10A—C11A—C12A—C13A	1.8 (11)	C10B—C11B—C12B—C13B	3.3 (12)
C18A—O5A—C13A—C14A	0.3 (12)	O4B—C12B—C13B—C14B	174.1 (8)
C18A—O5A—C13A—C12A	178.5 (7)	C11B—C12B—C13B—C14B	−4.5 (13)
O4A—C12A—C13A—O5A	1.0 (11)	O4B—C12B—C13B—O5B	−3.0 (11)
C11A—C12A—C13A—O5A	−177.7 (7)	C11B—C12B—C13B—O5B	178.4 (7)
O4A—C12A—C13A—C14A	179.3 (7)	C18B—O5B—C13B—C12B	179.2 (8)
C11A—C12A—C13A—C14A	0.5 (11)	C18B—O5B—C13B—C14B	2.2 (12)
O5A—C13A—C14A—C9A	175.3 (7)	C12B—C13B—C14B—C9B	4.6 (12)
C12A—C13A—C14A—C9A	−2.9 (11)	O5B—C13B—C14B—C9B	−178.5 (7)
C10A—C9A—C14A—C13A	3.0 (12)	C10B—C9B—C14B—C13B	−3.6 (12)
C8A—C9A—C14A—C13A	−175.6 (7)	C8B—C9B—C14B—C13B	−178.2 (7)

### Hydrogen-bond geometry (Å, º)

*Cg*1 and *Cg*2 are the centroids of the C9*A*–C14*A* and C9*B*–C14*B* rings, respectively.

**Table:**

*D*—H···*A*	*D*—H	H···*A*	*D*···*A*	*D*—H···*A*
N1*A*—H1*NA*···O1*A*^i^	0.86	2.11	2.944 (13)	164
N1*B*—H1*NB*···O1*B*^i^	0.85	2.21	2.939 (9)	144
C8*B*—H8*B*···O1*B*^i^	0.93	2.52	3.287 (11)	140
C15*B*—H15*D*···O2*A*^ii^	0.96	2.60	3.498 (14)	156
C17*B*—H17*D*···O4*B*^iii^	0.96	2.60	3.420 (10)	144
C16*A*—H16*B*···*Cg*1^iv^	0.96	2.66	3.429 (10)	138
C16*B*—H16*F*···*Cg*2^iv^	0.96	2.77	3.697 (10)	162
C18*A*—H18*B*···*Cg*1^i^	0.96	2.85	3.739 (10)	155
C18*B*—H18*F*···*Cg*2^i^	0.96	2.74	3.583 (10)	146

Symmetry codes: (i) *x*, *y*+1, *z*; (ii) −*x*+2, *y*+1/2, −*z*+1; (iii) −*x*+1, *y*−1/2, −*z*+2; (iv) *x*, *y*−1, *z*.
